# The Emerging Role of Exosomes in Cancer Chemoresistance

**DOI:** 10.3389/fcell.2021.737962

**Published:** 2021-10-28

**Authors:** Jing Li, Na Gao, Zhengfan Gao, Wei Liu, Bairen Pang, Xingli Dong, Yong Li, Tianli Fan

**Affiliations:** ^1^Department of Pharmacology, School of Basic Medical Science, Zhengzhou University, Zhengzhou, China; ^2^St George Hospital, St George and Sutherland Clinical School, Faculty of Medicine, UNSW Sydney, Kensington, NSW, Australia; ^3^Department of Biopharmaceutical Sciences, College of Pharmacy, Harbin Medical University, Harbin, China

**Keywords:** exosome, cancer, chemoresistance, liquid biopsy, biomarker

## Abstract

Chemoresistance is an impending challenge in cancer treatment. In recent years, exosomes, a subtype of extracellular vesicles with a diameter of 40–150 nm in bloodstream and other bio-fluids, have attracted increasing interest. Exosomes contain proteins, nucleic acids, and lipids, which act as important signaling molecules. Many reports indicate that exosomes play critical roles in chemoresistance through intercellular interactions, including drug removal from cells, transfer of drug resistance phenotypes to other cancer cells, and the increase in plastic stem cell subsets. Exosomes can reflect the physiological and pathological state of parent cells. Owing to their elevated stability, specificity, and sensitivity, exosomes are served as biomarkers in liquid biopsies to monitor cancer chemoresistance, progression, and recurrence. This review summarizes the exosome-mediated mechanisms of cancer chemoresistance, as well as its role in reversing and monitoring chemoresistance. The scientific and technological challenges and future applications of exosomes are also explored.

## Introduction

Chemotherapy is vital for cancer treatment. However, the majority of cancer patients develop drug resistance after repeated treatment, first to one and then to other chemotherapeutic agents (Gottesman, 2002). Chemoresistance is a major challenge for successful anticancer therapy. Cancer-derived exosomes have been implicated in chemoresistance by providing cancer cells with nucleic acids and proteins (Namee and O’Driscoll, 2018).

Exosomes are a subclass of heterogeneous extracellular vesicles (EVs) with a diameter of 40–150 nm, which are released from a variety of cells. Exosome secretion was initially considered as a form of waste excretion by cells (Johnstone, 1992). However, subsequent advances have revealed that exosomes play an important role in both physiological and pathological processes, highlighting their involvement in cell migration and invasion, immune response and chemoresistance (Namee and O’Driscoll, 2018; Yi et al., 2020). Exosomes are a key player in communicating among protein–protein, gene–gene, and gene–microRNA (miRNA) networks, as well as intracellular and distant cellular connection routes, which bestows them with untapped application potential (Kitano, 2004). Their role in cell communication suggests a potential connection between the dysregulation of exosomal cargo and chemoresistance in cancer. Increasing evidence indicates that exosomes mediate chemoresistance (Fan et al., 2018); therefore, exploring the underlying mechanism may help identify methods to prevent and reverse chemoresistance. In addition, exosomes hold great potential to be used as biomarkers and therapeutic tools in cancer in clinical settings (Xiao et al., 2020).

In this review, we focus on discussing the mechanisms of exosome-mediated chemoresistance in cancer, the use of exosomes to reverse chemoresistance, and their application as biomarkers in liquid biopsy to monitor chemoresistance. Finally, the technological challenges and future application prospects of exosomes in cancer therapy are also explored.

## Exosomes and Cancer Chemoresistance

### Exosomes in Cancer Research

Initially, exosomes were considered cellular waste and hence did not receive much attention. However, the characterization of nucleic acids, proteins, and lipids isolated from exosomes have advanced our understanding of the role of exosomes in intercellular communication and epigenetic regulation (Pathan et al., 2019). According to the ExoCarta database, over 9769 proteins, 3 408 mRNAs, and 2 838 miRNAs have been identified in exosomes to date. Exosomes have been found to play a vital role in mediating physiological processes and pathological conditions, wherein they mediate tumorigenesis, metastasis, angiogenesis, and drug resistance (Bach et al., 2017; Fan et al., 2018; Feng W. et al., 2019). Huang et al. (2019) demonstrated that prostate cancer-related transcript 1 (PCAT1) was present in esophageal squamous cell carcinoma (ESCC) cell-derived exosomes and promoted tumor cell growth through exosomes, and the level of exosomes was higher in the serum of ESCC patients than that in healthy volunteers. Liu X. et al. (2019) reported that miR-501 promoted tumorigenesis and chemoresistance in gastric cancer by targeting the BH3-like motif protein (BLID). As exosomes induce cancer occurrence and progression, these harmful processes can be prevented by inhibiting the production of exosomes or their uptake by target cells (Milman et al., 2019).

Under both physiological and pathological conditions, the content of exosomes is finely regulated by their parent cells, which transmit information to recipient cells and let them acquire specific functions. In turn, the functional status of parent cells can be estimated by analyzing exosome cargo. In general, exosomes act as a “double-edged sword”: on the one hand, they promote cancer progression; on the other hand, they target tumor cells with anticancer drugs. Consequently, exosomes are becoming effective tools for cancer diagnosis and treatment. In the following sections, we focus on the role of exosomes in cancer chemoresistance.

### The Role of Exosomes in the Tumor Microenvironment

The tumor microenvironment (TME) includes cancer cells, surrounding stromal cells (e.g., fibroblasts, inflammatory cells, and immune cells), cellular stroma, microvessels, and biomolecules. The TME is characterized by hypoxia, low pH, and high pressure. Due to these characteristics, the TME is rich in growth factors, cell chemokine factors, and proteolytic enzymes, which facilitate cancer proliferation, invasion, adhesion, angiogenesis, and chemoresistance (Cairns et al., 2006; Hui and Chen, 2015). For a long time, scientists focus exclusively on cancer cells and the use of targeted drugs to inhibit their growth. However, accumulating studies have shown that the TME plays an essential role in cancer invasion, metastasis, and chemoresistance (Hui and Chen, 2015; Ren et al., 2018). Ombrato et al. (2019) discovered that cancer-associated parenchymal cells in the TME exhibited stem cell-like characteristics, which helps cancer cells survive, spread, and develop resistance to treatment.

Exosomes provide a key communication channel between the TME and cancer cells. Wu et al. (2019) demonstrated that exosomes in the TME promoted the occurrence and progression of hepatocellular carcinoma by regulating energy metabolism and the inflammation microenvironment, as well as by inducing angiogenesis. Zhang X. et al. (2019) found that specific miRNAs carried by exosomes, including miR-193-3p, miR-210-3p, and miR-5100, promoted invasion by lung cancer cells through activation of the STAT3-induced epithelial-to-mesenchymal transition (EMT). Non-tumor cells in the TME were reported to be an important source of drug resistance, providing factors that facilitate cancer cell survival even during chemotherapy (Wilson et al., 2012). Xu et al. (2019) found that lncPSMA3-AS1 and PSMA3 were packaged into exosomes and transferred from mesenchymal stem cells to multiple myeloma, promoting proteasome inhibitor resistance (Xu et al., 2019). Binenbaum et al. (2018) shown that miR-365 in macrophage-derived exosomes inhibited the activation of gemcitabine by increasing nucleotide triphosphate levels and inducing cytidine deaminase, causing pancreatic cancer cells to become resistant to gemcitabine. From a clinical point of view, the identification of soluble resistance factors such as exosomes helps overcome drug resistance in cancer cells. This can be achieved by preventing exosomes from exerting protective effects on cancer cells and stimulating, instead, chemosensitivity.

### The Mechanism Underlying Exosome-Mediated Chemoresistance

**FIGURE 1:**
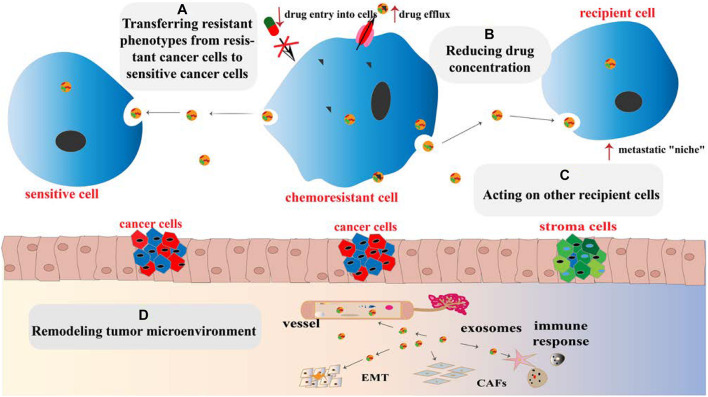
Mechanisms for promoting chemoresistance by exosomes. **(A)** Exosomes transfer chemoresistant phenotypes from resistant cancer cells to sensitive cancer cells. **(B)** The intracellular drug concentration can be reduced by increasing drug efflux, reducing cell lysis, and isolating cytotoxic drugs via exosomes. **(C)** Exosomes acting on recipient cells induce the formation of a “niche” through transferring their contents and recoding cell cycle and apoptosis genes of recipient cells. **(D)** Exosomes remodel the TME by increasing cancer cell immune escape, promoting cancer angiogenesis, EMT, and transforming basal fibroblasts into CAFs or even cancer cells.

Chemoresistance is divided into primary drug resistance and multiple drug resistance (MDR). The former refers to cancer cells that are resistant to induced drugs, whereas the latter refers to cancer cells that develop resistance to induced drugs, as well as to other chemotherapeutic agents with different structures and mechanisms, to which cells were not even exposed (Gacche and Assaraf, 2018). The establishment of chemoresistance in cancer cells involves a variety of mechanisms, including increased DNA repair, downregulation of apoptosis, altered drug targets, increased drug efflux, and overexpression of MDR proteins (Housman et al., 2014; Alharbi et al., 2018). Growing evidence has demonstrated the role of exosomes in mediating cancer chemoresistance (Figure 1), and the following underlying mechanisms are proposed:

(1) Influencing recipient cells: exosomes induce the formation of the pre-metastatic niche between the primary cancer and remote organs and recode cell cycle and apoptosis genes of recipient cells in the TME. Organ-specific metastasis is guided by the adhesion molecules such as integrins on the surface of exosomes (Hoshino et al., 2015).

(2) Limiting effective drug utilization by regulating drug efflux, reducing cell cytolysis, and isolating cytotoxic drugs (Pilzer and Fishelson, 2005).

(3) Transferring chemoresistance phenotypes from drug-resistant to sensitive cells (Milman et al., 2019).

(4) Reshaping the TME: exosomes increase cancer cell immune escape, promote local cancer angiogenesis and EMT, and transform basal fibroblasts into cancer-associated fibroblasts (CAFs) or even cancer cells through TME remodeling (Wang et al., 2018).

The exosome proteins and nucleic acids mediate signal transduction and regulate gene expression by initiating signal transduction pathways or binding to cell surface receptors. We will discuss hereafter how proteins and nucleic acids in exosomes mediate chemoresistance.

#### Proteins in Exosome-Mediated Chemoresistance

Exosomes have been shown to transfer proteins, such as *P*-glycoprotein (*P*-gp), annexin A3, ATPase copper transporting alpha (ATP7A), ATPase copper transporting beta (ATP7B), and survivin, from drug-resistant cells to sensitive cells or to induce direct drug efflux (Table 1). All these processes result in chemoresistance in sensitive cancer cells.

**TABLE 1:** Proteins associated with exosome-mediated chemoresistance in cancer.

Cancer type	Protein	Drug-mediated resistance	References
Breast cancer	*P*-gp	Adriamycin	Ning et al., 2017
	*P*-gp	Docetaxel	Lv et al., 2014
	Survivin	Paclitaxel	Kreger et al., 2016
Ovarian cancer	DNMT1	Cisplatin	Cao et al., 2017
	*P*-gp	Paclitaxel	Zhang et al., 2014
	Annexin A3	Platinum	Yan et al., 2010; Yin et al., 2012
	ATP7A/ATP 7B/MRP1	Cisplatin	Safaei et al., 2005
Leukemia	*P*-gp	Multidrug	Bebawy et al., 2009
	MRP1	Multidrug	Lu et al., 2013
Prostate cancer	*P*-gp	Docetaxel	Kato et al., 2015
Colorectal cancer	*P*-STAT3	5-Fluorouracil	Zhang Q. et al., 2019
Pancreatic cancer	EphA2	Gemcitabine	Fan et al., 2018
Diffuse large B cell lymphoma	CA1	R-CHOP	Feng et al., 2020

*ATP7A/ATP7B, ATPase copper transporting alpha/beta; CA1, carbonic anhydrase 1; DNMT1, DNA methyltransferase 1; EphA2, ephrin type-A receptor 2; MRP1, multidrug resistance protein 1; *P*-gp, *P*-glycoprotein; R-CHOP, cyclophosphamide, doxorubicin, vincristine, and prednisone, combined with rituximab.*

*P*-gp is a 170-kDa protein encoded by the MDR-1 gene and plays a key role in maintaining appropriate intracellular drug concentration. It is currently one of the most studied proteins in cancer chemoresistance (Bebawy et al., 2009; Lv et al., 2014; Zhang et al., 2014; Kato et al., 2015). Ning et al. (2017) demonstrated that in adriamycin (ADM)-resistant human breast cancer cells (MCF7/ADM), ADM did not accumulate in the nuclei as expected but was secreted from the cells through exosomes. At the same time, a high level of *P*-gp was observed in the exosomes, together with the increased levels of its regulator transient receptor potential channel 5 (TrpC5). *P*-gp was found to be restored using a TrpC5 channel-specific blocker (T5E3), which limited drug efflux and increased breast cancer chemosensitivity (Ning et al., 2017). Further studies demonstrated that exosomes from MCF7/ADM cells carried ubiquitin carboxyl terminal hydrolase L1 (UCH-L1) and P-gp into the extracellular microenvironment, enabling their time-dependent integration in ADM-sensitive human breast cancer cells (MCF7/WT) and acquisition of drug-resistant phenotypes. Furthermore, the level of UCH-L1 in blood exosomes of breast cancer patients was found to correlate negatively with their prognosis. This can be explained by overexpression of UCH-L1, enhancing MDR in breast cancer through activation of the MAPK/ERK signaling pathway and upregulation of *P*-gp (Ning et al., 2017). In another study, the level of *P*-gp was reported to be significantly higher in exosomes isolated from docetaxel-resistant prostate cancer cells than in parent cells (Kato et al., 2015). The same result was observed in clinical blood samples, indicating that exosomal P-gp was associated with docetaxel resistance in prostate cancer and holds potential to be used as a biomarker to monitor docetaxel-resistant prostate cancer in clinic (Kato et al., 2015).

Annexin A3 belongs to the phospholipid-binding protein family, which is involved in intercellular interactions and is closely related to cancer cell invasion, metastasis, and chemoresistance (Donnelly and Moss, 1997; Yan et al., 2010; Yin et al., 2012). Yan et al. (2010) reported that annexin A3 was significantly enriched in Pt-resistant ovarian cancer cell lines, where it reduced the concentration of intracellular Pt-based drugs and prevented cancer cell apoptosis. Further studies showed that upregulation of annexin A3 in cancer cells led to the formation and release of multivesicular body vesicles. Western blotting and immunoelectron microscopy further confirmed the presence of annexin A3 in exosomes, suggesting that the latter transferred annexin A3 between cells to induce chemoresistance (Yan et al., 2010).

Ubiquitin proteins (survivin), DNA methyltransferase 1 (DNMT1), copper transporters (ATP7A and ATP7B), multidrug resistance protein (MRP2), P-STAT3, and carbonic anhydrase 1 (CA1) in exosomes have also been reported to induce chemoresistance (Safaei et al., 2005; Lu et al., 2013; Kreger et al., 2016; Cao et al., 2017; Zhang Q. et al., 2019; Feng et al., 2020). Survivin is a key factor in maintaining apoptosis resistance, and was frequently reported to be involved in cancer multidrug resistance (Hu et al., 2015; Han et al., 2016; Wang et al., 2016). Kreger et al. (2016) found that survivin was significantly enriched in paclitaxel-resistant breast cancer cells, where it promoted cell survival and drug resistance, and the effect that was ablated when survivin was knocked down from these exosomes. Aspe et al. (2014) reported that the dominant-negative mutant of survivin (Survivin-T34A) was shown to block survivin, inducing caspase activation and apoptosis, and enhancing the sensitivity of gemcitabine in pancreatic adenocarcinoma. Both epigenetic alterations and genetic variations play critical roles in tumorigenesis and development (Feinberg et al., 2016). DNMT1 is a key gene for DNA methylation in epigenetic modification, and the protein encoded by it is a complex enzyme with multiple regulatory functions in cancer (Ren et al., 2020). Cao et al. (2017) demonstrated that DNMT1 transcripts were significantly enriched in ovarian cancer cell lines. After the *in vivo* administration of cisplatin, DNMT1-containing exosomes were found to exacerbate xenograft progression and significantly reduce overall survival (Cao et al., 2017). The multidrug resistance of cancer cells is mainly caused by membrane protein ATP binding cassettes (ABCs), which excretes toxic substances from the body. After drug treatment, the expression of various genes involved in cells transport increased, such as ATP7A, ATP7B, and MRP2. Likewise, Safaei et al. (2005) reported greater secretion of exosomes in cisplatin-resistant ovarian cancer cell lines (2008/C13^∗^5.25) than in sensitive cells, together with significant upregulation of ATP7A, ATP7B, and MRP2. More and more studies have reported that CA1 is related to cancer progression. Feng et al. showed that compared to diffuse large B cell lymphoma (DLBCL) parental cells, the expression of CA1 is upregulated in chemoresistant cells, and the expression level of CA1 was higher in exosomes from the blood of chemoresistant patients compared with chemosensitive patients (Feng et al., 2020). Knockdown of CA1 inhibited the growth of DLBCL cells via inhibiting the activation of the NF-kB and STAT3 signaling pathways both *in vitro* and *in vivo*. The researcher proposed that exosomal CA1 predicted prognosis and chemotherapeutic efficacy in DLBCL patients (Feng et al., 2020). Several preclinical and clinical studies have demonstrated that STAT3 is activated in many cancers and correlates with patient’s survival (Birner et al., 2010). Moreover, it has been reported increased p-STAT3 levels are associated with chemoradiotherapy (Spitzner et al., 2014). Zhang X. et al. (2019) demonstrated that exosomal transfer of p-STAT3 promoted acquired 5-FU resistance in colorectal cancer cells. In summary, a growing body of evidence shows that the proteins in exosomes induce chemoresistance in sensitive cells through various mechanisms, such as the transmission of chemoresistance phenotypes and reduction of intracellular drug concentrations. It is worth noting that these proteins show promise as biomarkers for the monitoring of chemoresistance.

#### Nucleic Acids in Exosome-Mediated Chemoresistance

Nucleic acids in exosomes comprise DNA and RNA fragments. In recent years, several studies have demonstrated that exosomal miRNAs regulate intracellular RNA and protein levels, playing a crucial role in cancer chemoresistance (Patel et al., 2017; Qin et al., 2019).

Exosomes secreted by drug-resistant cancer cells act as paracrine regulators by transferring genetic material (O’Brien et al., 2013). The miRNAs found in exosomes secreted by chemoresistant cancer cells deliver drug-resistant phenotypes to sensitive cancer cells, thereby influencing cell growth and inducing anti-apoptotic processes (Table 2; Chen et al., 2014; Jin et al., 2015; Pink et al., 2015; Weiner-Gorzel et al., 2015; Au et al., 2016; Bouvy et al., 2017; Qin et al., 2017; Wei et al., 2017; Fu et al., 2018; Kanlikilicer et al., 2018; Min et al., 2018; Zhang Y. et al., 2018; Zhu et al., 2019; Han et al., 2020). Moreover, miR-501 is upregulated in lung adenocarcinoma, cervical cancer, liver cancer, and gastric cancer, suggesting that miR-501 is a carcinogenic miRNA (Fan et al., 2016; Xu et al., 2018; Liu X. et al., 2019). Liu X. et al. (2019) found that miR-501 promoted tumorigenesis and chemoresistance of gastric cancer by targeting BLID. Compared with exosomes (7901 Exo) secreted by sensitive gastric cancer cell lines (SGC7901), miR-501 expression was significantly upregulated in exosomes (ADR Exo) secreted by doxorubicin-resistant cell lines (SGC7901/ADR). After ADR Exo were ingested by SGC7901 cells, miR-501 was incorporated, increasing chemoresistance of gastric cancer cells to doxorubicin. Knockdown of miR-501 and overexpression of BLID, via exosome inhibitors (GW4869) or miR-501 inhibitors, restored the sensitivity of chemoresistant cells to doxorubicin and inhibited the proliferation, migration, invasion, and apoptosis of gastric cancer cells (Liu X. et al., 2019). In addition to exosomes derived from cancer cells, exosomes originating from stromal cells are also involved in cancer resistance. Hu et al. (2019) reported that CAFs directly transferred exosomes to colorectal cancer (CRC) cells and upregulated miR-92a-3p expression. In this way, CAFs were able to promote stemness, EMT, chemoresistance, and metastasis of CRC (Hu et al., 2019). In clinical studies, high expression of exosomal miR-92a-3p in serum was found to be closely related to metastasis and chemoresistance in CRC patients; hence, miR-92a-3p in exosomes secreted by CAFs is a useful biomarker for monitoring the progression of CRC (Hu et al., 2019).

**TABLE 2:** miRNAs associated with exosome-mediated cancer chemoresistance.

Cancer type	miRNAs	Drug-mediated resistance	References
Lung cancer	miR-222-3p	Gemcitabine	Wei et al., 2017
	miR-214	Gefitinib	Zhang Y. et al., 2018
	miR-100-5p	Cisplatin	Qin et al., 2017
Breast cancer	miR-222	Docetaxel	Chen et al., 2014
	miR-567	Truzhuo	Han et al., 2020
	miR-155-3p	Paclitaxel	Au et al., 2016
Ovarian cancer	miR-21	Paclitaxel	Au et al., 2016
	miR-433	Paclitaxel	Weiner-Gorzel et al., 2015
	miR-21-3p	Cisplatin	Pink et al., 2015
	miR-1246	Paclitaxel	Kanlikilicer et al., 2018
	miR-223	Cisplatin	Zhu et al., 2019
Leukemia	miR-365	Imatinib	Min et al., 2018
	miR-19b	Multidrug	Bouvy et al., 2017
	miR-20a	Multidrug	Bouvy et al., 2017
Hepatocellular cancer	miR-32-5p	Multidrug	Fu et al., 2018
Gastric cancer	miR-21	Paclitaxel	Jin et al., 2015
	miR-501	Doxorubicin	Fan et al., 2016; Xu et al., 2018; Liu X. et al., 2019
	miR-522	Cisplatin and Paclitaxel	Zhang et al., 2020
	miR-374-5p	Oxaliplatin	Ji et al., 2019
Oral cancer	miR-21	Cisplatin	Liu et al., 2017
B-cell lymphoma	miR-99a-5p	Doxorubicin	Feng Y. et al., 2019
	miR-125b-5p	Doxorubicin	Fan et al., 2016
Colorectal cancer	miR-92a-3p	5-FU	Hu et al., 2019
Head and neck cancer	miR-196-a	Cisplatin	Qin et al., 2019
Pancreatic cancer	miR-365	Gefitinib	Binenbaum et al., 2018
	miR-210	Gefitinib	Binenbaum et al., 2018
	miR-155	Gefitinib	Patel et al., 2017

*5–FU, 5-fluoro-2,4(1H,3H)-pyrimidinedione.*

In addition to miRNAs, other nucleic acids, such as long non-coding RNAs (lncRNAs) and circular RNAs (circRNAs), are also responsible for inducing chemoresistance. Qu et al. (2016) reported that during sunitinib treatment of renal cancer, the content of lncARSR in exosomes started to increase concomitantly with the development of drug resistance. Further studies found that lncARSR induced sunitinib resistance mainly through the competitive binding of miR-34/miR-449, which increased the expression of receptor tyrosine kinases (AXL and c-MET) in renal cancer cells, while promoting chemosensitivity via lncARSR inhibitors (Qu et al., 2016). Kang et al. (2018) elucidated that lncRNA PART1 was upregulated in gefitinib-resistant esophageal squamous cell carcinoma (ESCC) cells, where it regulated the miR-129/Bcl-2 pathway. Importantly, knockout of lncRNA PART1 effectively promoted gefitinib-induced cell death. Extracellular PART1 was found to enter exosomes and spread to sensitive cells, thereby inducing gefitinib resistance (Kang et al., 2018). Studies on clinical samples have shown that high levels of serum lncRNA PART1 in exosomes are associated with a poor prognosis in ESCC patients treated with gefitinib (Kang et al., 2018).

Some circRNAs are also shown to function as effective endogenous miRNA competitive molecules and have the potential to participate in cancer gene regulation. Based on both *in vitro* and *in vivo* evidence, Wang et al. (2020) demonstrated that exosomes secreted by oxaliplatin-resistant cells transferred circRNA-122 to drug-sensitive cells, wherein they enhanced glycolysis and drug resistance by upregulating the M2 isoform of pyruvate kinase and reducing miR-122 level. Knockdown of circRNA-122 was found to inhibit glycolysis and reverse drug resistance (Wang et al., 2020). Studies have also shown that mitochondrial DNA in exosomes regulates the immune escape of hormone-resistant breast cancer cells and induces chemoresistance (Sansone et al., 2017).

In summary, many preclinical and clinical studies have demonstrated that nucleic acids in exosomes play a vital role in mediating chemoresistance, leading to an increased interest in miRNA research. Inhibiting or inducing the expression of these nucleic acids can reverse chemotherapy resistance; whereas monitoring their levels can prevent chemoresistance and increase overall survival.

## Strategies to Combat Exosome-Mediated Chemoresistance

To maximize the effect of chemotherapy, it is essential to inhibit exosome-associated chemoresistance. To this end, two possible strategies exist: (i) inhibition of exosome biogenesis and (ii) application of exosomes as delivery vehicles.

### Inhibition of Exosome Biogenesis

Because exosomes induce chemoresistance in cancer cells, several studies have hypothesized that inhibition of exosome biogenesis may reverse chemoresistance. Inhibiting the formation or release of multivesicular bodies (MVBs) is the most important way to inhibit the biogenesis of exosomes (Essandoh et al., 2015; Sterzenbach et al., 2017). Hydrochloride hydrate (GW4869), a commonly used drug to inhibit the formation of exosomes, was reported to block the sprouting of MVBs mediated by ceramide, thereby inhibiting the release of exosomes from MVBs (Luberto et al., 2002). Luberto et al. (2002) found that after GW4869 treatment of cells, the exosomal proteolipid protein (PLP) and CD63 in the ultracentrifugation component were significantly reduced. Richards et al. (2017) reported that gemcitabine treatment of CAFs caused an increase in the release of chemoresistance-promoting exosomes. Treatment of gemcitabine-exposed CAFs with GW4869 significantly reduced cells survival, indicating the important role of GW4869 in reversing chemoresistance (Richards et al., 2017). Furthermore, Manumycin-A, a natural microbial metabolite, was identified as an inhibitor of exosome biogenesis and secretion in castration-resistant prostate cancer (CRPC) without affecting cell growth. Datta et al. (2017) showed that, in CRPC cells, manumycin-A suppressed exosome biogenesis and secretion via the targeted inhibition of Ras/Raf/ERK1/2 signaling and ERK-dependent inhibition of the oncogenic splicing factor hnRNP H1. Using a CRC xenograft mouse model, Gu et al. (2020) showed that neticonazole acted as a promising exosome secretion inhibitor, suppressing intestinal dysbacteriosis-induced tumorigenesis of CRC. Similarly, the exosome blocker indomethacin causes doxorubicin to effectively accumulate in the cell nuclei, instead of expelling it via exosomes. This strategy improved cytotoxicity and cell retention of the drug, thereby reversing chemoresistance (Araujo et al., 2016).

Reversing chemoresistance by inhibiting the biogenesis of exosomes is promising but challenging. Im et al. (2019) reported that the antibacterial drug sulfisoxazole exerted an antitumor effect by inhibiting the secretion of breast cancer cell exosomes. However, Fonseka et al. (2021) recently found that treating cancer cells with sulfisoxazole did not reduce the number of exosomes. This may be due to discrepancies between the two studies, such as cell lines from different passages or different equipment. Overall, drug incubation studies point to the inhibition or promotion of exosome release. This strategy is still in its infancy and requires more extensive experimental and clinical verifications in the future.

### Application of Exosomes as Delivery Vehicles

In recent decades, synthetic nanoparticles (NPs) including liposomes, nanosponges, and self-assembling peptides have been widely studied for nanomedicine, especially targeted cancer therapy (Allahyari et al., 2019; Crommelin et al., 2020; Wang et al., 2021). Due to the heterogeneity of target cancer cells, the differences in biological barriers and immune systems between human and animal models, exogenous nanomaterials used to deliver drugs to target sites are facing many obstacles (Ben-David et al., 2017). One approach to overcome the limitations of synthetic NPs is developing natural carriers. Extensive evidence shows that exosomes are able to pass through lipid bilayer cell membranes, aided by their low immunogenicity, high biocompatibility, high delivery efficiency, and good stability in circulation. This property supports the potential of exosomes in drug or gene delivery.

Packing exosomes with small molecule chemotherapeutic drugs have been increasingly reported. Since the chemoresistant cells easily flush out the drugs, researchers collected the supernatant of the chemoresistant cells treated with paclitaxel and centrifuged to obtain paclitaxel-loaded exosomes to simply complete the drug loading (Yamagishi et al., 2013). It is worth mentioning that, loading drugs into exosomes improved drug stability and bioavailability and maximized their effect on target lesions. For example, Kim et al. (2016) demonstrated that, in drug-resistant cells, the incorporation of paclitaxel into exosomes increased cytotoxicity by more than 50 times, with carrier exosomes co-localizing almost completely with cancer cells. Although exosomes as delivery vehicles have achieved encouraging results in previous studies, the clinical transformation of exosomes is being challenged by large-scale production, purification, drug loading and storage. In addition, the heterogeneity between exosomes subgroups greatly hinders the quality control of manufacturing and clinical translation. In view of the shortcomings of natural exosomes, more and more researchers have developed artificial exosomes through nanobiotechnology, which brought great hope for the advanced drug delivery combined with the advantages of natural and synthetic NPs (Li et al., 2021). Given that the dissemination of exosomal cargo mediates the development of chemoresistance in tumor cells, exploring the exosomal contents may open a new avenue for the reversal of chemoresistance. Wu et al. (2020) found that upregulation of miR-193a in exosomes derived from bone marrow mesenchymal stem cells reduced cisplatin resistance of non-small cell lung cancer cells by targeting leucine-rich repeat containing 1. To improve the efficacy of cancer treatment, the above two strategies were combined. For example, miR-21 can induce 5-fluoro-2,4(1H,3H)-pyrimidinedione (5-FU) resistance in CRC cells. Liang et al. (2020) used electroporation to load miR-21 inhibitor and 5-FU into exosomes to generate a co-delivery system (THLG-EXO/5-FU/miR-21i). The latter effectively reversed drug resistance and significantly enhanced cytotoxicity in chemoresistant cells. Therefore, the successful delivery of conventional drugs and various genetic materials by exosomes offers a promising strategy for reversing chemoresistance.

## Use of Exosomes as Biomarkers in Liquid Biopsy to Monitor Chemoresistance

Exosomes contain a series of biomolecules, including membrane-bound proteins and soluble proteins, miRNAs, non-coding RNAs, and lipids, which are good sources of biomarkers for cancer diagnosis and monitoring of cancer progression. Liquid biopsy allows the detection of cancer or other biomarkers in blood, urine, and other body fluids. It serves to detect the occurrence of diseases, tracks cancer progression, and predicts chemoresistance (Johann et al., 2018). Compared with traditional cancer diagnosis methods, liquid biopsy improves diagnostic precision, reduces the harm and discomfort of solid tissue biopsy, and potentially prolongs patients’ survival. Commonly, liquid biopsy includes detecting circulating tumor cells (CTCs), circulating tumor DNA (ctDNA), and exosomes (Nimir et al., 2019). The use of exosomes for liquid biopsy has attracted growing attention owing to (1) high sensitivity and specificity compared to that of traditional methods, (2) detection in various body fluids via non-invasive methods, (3) high stability and durability in the extracellular environment, and (4) an indication of the physiological and pathological state of parent cells. Therefore, the use of exosomal biomarkers represents an important clinical advancement in precision medicine (Tai et al., 2018). Indeed, some diagnostic kits based on exosomes have been approved by the US Food and Drug Administration (FDA) and are currently being used for clinical diagnosis (Xiao et al., 2020).

Although promising results have been achieved in targeted cancer therapy and immunotherapy, the gradual development of chemoresistance poses a significant risk to patients’ overall survival. In addition to understanding the mechanism of drug resistance and overcoming chemoresistance, the identification of suitable biomarkers helps adjust drug regimens at the earliest sign of resistance. Accumulating evidence shows that nucleic acids and proteins in exosomes are useful biomarkers for the detection of drug resistance in patients during chemotherapy (Table 3; Lobb et al., 2017; Lei et al., 2018; Zhang W. et al., 2018). In the quest for chemoresistance biomarkers, exosomes have been extracted from cancer cell lines, animal models, and clinical samples. To this end, high-throughput proteomics and genomics have been performed to identify proteins and/or RNAs expressed differently in exosomes secreted by chemosensitive vs. chemoresistant cells. Future experiments will determine whether certain proteins and RNAs are suitable to be used as potential biomarkers of chemoresistance in clinical settings, thus providing new strategies for targeted cancer treatment.

**TABLE 3:** Potential exosome molecular biomarkers used in chemoresistance.

Cancer type	Exosome biomarker	Drug-mediated resistance	Investigation model	References
Lung cancer	RNA-based: miR-222-3p	Gemcitabine	A549-GR and A549-P cell lines, female severe combined immunodeficient (SCID) mice (*n* = 12), and human blood samples (*n* = 50)	Wei et al., 2017
	RNA-based: miR-214	Gefitinib	PC-9 and PC-9GR cell lines and male BALB/c nu/nu mice (*n* = 20)	Zhang Y. et al., 2018
	RNA-based: miR-100-5p	Cisplatin	A549 and A549/DDP cells lines and BABL/c athymic nude mice (*n* = 25)	Qin et al., 2017
	RNA-based: mRNA ZEB1	Multidrug	HBEC and HBECs with p53 knockdown, KRASV12 overexpression, and LKB1 knockdown cell lines	Lobb et al., 2017
	RNA-based: lncRNA H19	Gefitinib	HCC827 and HCC4006 cell lines	Lei et al., 2018
	RNA-based: lncRNA RP11-838N2.4	Erlotinib	HCC827, HCC827/R, HCC4006, and HCC4006/R cells line and serum samples (*n* = 78)	Zhang W. et al., 2018
	RNA-based: miRNA-146a-5p	Gemcitabine	Serum samples (*n* = 100) and paraffin sections of lung cancer tissues (*n* = 12)	Yuwen et al., 2017
	RNA-based: miRNA-425-3p	Cisplatin	A549, PC-9, SPCA1, H1299, H1650, H1703, H1975, and A549/DDP cell lines, serum samples (*n* = 114), and paraffin sections of lung cancer tissues (*n* = 203)	Yuwen et al., 2019
	RNA-based: lncRNA SNHG14	Trastuzumab	SKBR-3, BT474, SKBR-3/Tr, and BT474/Tr cell lines and serum samples (*n* = 72)	Dong et al., 2018
	Protein-based: *P*-gp	ADM/Docetaxel	MCF7/WT and MCF7/ADM cell lines and plasma samples (*n* = 93)	Ning et al., 2017
	Protein-based: HER2	Trastuzumab	SKBR3, BT474, and MDA-MB-231 cell lines	Ciravolo et al., 2012
	RNA-based: mRNA UCH-L1	ADM	MCF7/WT and MCF7/ADM cell lines and plasma samples (*n* = 93)	Ning et al., 2017
Ovarian cancer	RNA-based: miR-1246	Paclitaxel	HeyA8, SKOV3-ip1, A2780, HeyA8-MDR, SKOV3-TR, A2780-CP20, THP-1, and HIO180 cell lines and female athymic nude mice (*n* = 40)	Kanlikilicer et al., 2018
	Protein-based: *P*-gp	Paclitaxel	A2780, SKOV3, MCF-7, MBA-MD-231, Hel, and THP1 cell lines and female athymic nude mice (*n* = 30)	Zhu et al., 2019
	Protein-based: Annexin A3	Platinum	SKOV3, SKOV3/Cis, SKOV3/Car, A2780, A2780/Cis, and A2780/Car cell lines and tissue samples (*n* = 42)	Yan et al., 2010
Hepatocellular cancer	RNA-based: miR-32-5p	Multidrug	Bel7402, Bel/5-FU, HEK-293T, SMCC-7721, HepG2, Hep3B, and MHCC97H cell lines and tissues samples (*n* = 72)	Fu et al., 2018
	RNA-based: linc-ROR	Doxorubicin/Camptothecin	HepG2, PLC-PRF5, and HH cell lines	Takahashi et al., 2014
	RNA-based: lnc-VLDLR	Multidrug	HepG2, Hep3B, PLC/PRF-5, Huh-7, and MzChA-1 cell lines	Takahashi et al., 2014
	RNA-based: miR-744	Sorafenib	LO2 cell line and tissue samples (*n* = 120)	Wang et al., 2019

*ADM, adriamycin; HER2, human epidermal growth factor receptor-2.*

### Exosomal Contents as Diagnostic Biomarkers for Chemoresistance

Both preclinical and clinical studies have reported the use of exosomes as biomarkers to predict cancer chemoresistance in liquid biopsy. Yang S. J. et al. (2017) demonstrated that exosomes transferred glutathione S-transferase P1 (GSTP1) from breast cancer resistant cells to sensitive cells, thereby increasing resistance to ADM. Therefore, GSTP1 can be used as a biomarker to predict anthracycline/taxane-based chemoresistance in breast cancer treatment. Hu et al. (2019) reported the elevated miR-92a-3p expression in exosomes secreted by CAFs in a study of metastatic and chemoresistant CRC patients. These exosomes promoted cancer metastasis and chemoresistance by increasing stem cell subsets and EMT of CRC cells. Accordingly, these miRNAs in CAF-exosomes hold potential as biomarkers to monitor cancer cell proliferation and chemoresistance (Hu et al., 2019).

### Exosomes From Different Body Fluids as Biomarkers for Chemoresistance Diagnosis

**FIGURE 2:**
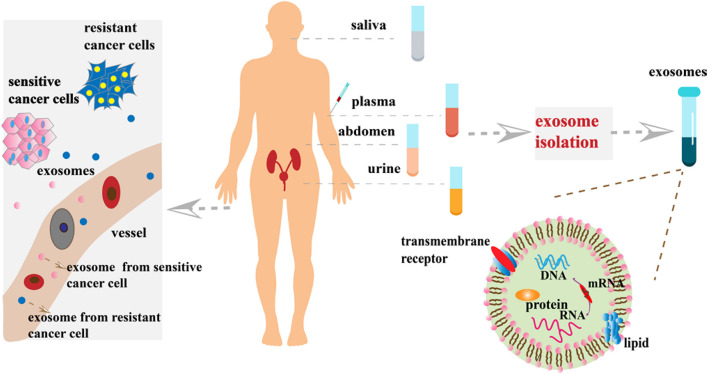
Exosome-based analytes of liquid biopsies. At the primary cancer site, resistant cancer cells and sensitive cancer cells secrete large numbers of exosomes into the blood stream and/or invade adjacent blood vessels. Exosomes present in various body fluids (e.g., plasma, saliva, and urine) can then be separated by various methods for cancer diagnosis and monitoring progression.

In recent years, the use of exosomal biomarkers in urine and blood samples has become more widespread. Exosomes in urine are often used as biomarkers for the detection of prostate, bladder, kidney, and other urinary system cancers. Comparing lipid composition of urine exosomes prostate cancer patients with normal healthy subjects, Skotland et al. (2017b) found that such lipids are useful biomarkers to diagnose and track the progress of prostate cancer. Xu et al. (2019) found that the exosomes containing lncPSMA3 and proteasome subunit α7-encoding PSMA3-AS1 promoted resistance to protease inhibitors by multiple myeloma. The newly identified PSMA3/PSMA3-AS1 signaling pathway in exosomes may serve as a new therapeutic target for protease inhibitor resistance or as a biomarker for the detection of chemoresistance (Xu et al., 2019). However, blood contains a large number of proteins, making the detection of poorly expressed biomarkers difficult. Unlike blood, exosomes in saliva can be easily detected. Sun et al. (2018) identified 785 highly stable and biologically active proteins in salivary exosomes. They also demonstrated that salivary exosomal proteins could be used for the non-invasive detection of lung cancer (Sun et al., 2018). Blood-derived molecules can enter salivary glands through a transcellular or cell bypass, suggesting the use of exosomes in saliva as biomarkers of cancer progression (Yoshizawa et al., 2013). Lin et al. (2019) discussed the potential clinical application of GOLM1-NAA35 chimeric RNA (seG-NchiRNA) from salivary exosomes in ESCC and found that it could help monitor tumorigenesis and sensitivity. Exosomes in urine, blood, and saliva are useful sources as biomarkers, whereas exosomes in other body fluids, such as ascites, breast milk, and cerebrospinal fluid, can also serve as biomarkers in liquid biopsy to monitor cancer chemosensitivity (Figure 2).

## Current Challenges and Future Prospects

### The Challenges of Applying Exosomes in Cancer Chemoresistance

At present, research on the mechanism of exosome-mediated chemoresistance is still in its infancy. The lack of biomarkers for predicting chemoresistance and methods to reverse resistance is one of the greatest challenges facing the field of cancer research. In clinical practice, further research is needed on the proteins and nucleic acids used as biomarkers to diagnose drug resistance in patients. However, the most important challenges are two-fold: (i) a lack of standardized methods for the separation and purification of exosomes and (ii) the difficulty in quantifying exosomes in different body fluids.

According to the 2015 Global International Society for Extracellular Vesicles (ISEV) survey, ultracentrifugation, polymer precipitation, immunomagnetic beads, and size exclusion chromatography are the most commonly used exosome separation techniques. However, these methods have several limitations, including cumbersome and complex operational steps, the need for expensive equipment, and low separation purity (Gardiner et al., 2016). Although ultracentrifugation is the “gold standard” for exosome separation, it causes protein contamination (Caradec et al., 2014). According to the ISEV 2018 guidelines, to achieve better specificity in exosome separation, more than one technique should be applied after the primary step, including chromatography, washing in EV-free buffer, ultrafiltration, or the application of density gradients (Thery et al., 2018). The emergence of new technologies is providing novel solutions for efficient exosome separation. Microfluidic technology has attracted attention due to its high speed, high purity, short time, and ability to process small-volume samples (Yang F. et al., 2017). Currently, microfluidic exosome separation methods comprise three categories: size-based separation, immunoaffinity-based separation, and dynamic exosome separation. Immunoaffinity-based microfluidic exosome separation has been successful for the clinical detection of cancers, thus providing a new analysis platform for cancer diagnosis and molecular typing (Yang F. et al., 2017). However, although microfluidic-based exosome separation methods have great potential, they are still in the early stages of development.

The exact quantification of exosomes remains a challenge, among other things, because their average concentration varies across body fluids (e.g., plasma, saliva, urine, and lymph). Eitan et al. (2017) determined that the average concentration of exosomes isolated from human plasma was between 1 × 10^9^ and 3 × 10^12^ particles per mL. Similarly, the average concentration of exosomes was estimated to be between 2 × 10^8^ and 1 × 10^9^ in urine and between 1 × 10^11^ and 2 × 10^12^ particles per mL in lymph (Eitan et al., 2017; Gheinani et al., 2018; Broggi et al., 2019). Most clinical studies are based on very small sample sizes. However, biomarkers must be validated in large sample size studies before they are converted into clinical application tools. This requires highly specific and sensitive surface markers, which are difficult to identify for most cancer types. Another solution is to select different body fluids for exosome detection based on cancer type. Compared with peripheral blood, the pulmonary vein blood of lung cancer patients is significantly enriched with tumor-derived exosomes (Navarro et al., 2019). Similarly, Matsumura et al. (2019) demonstrated that tumor-derived exosomes were significantly enriched in the lymph compared to the plasma of melanoma patients, and lymphatic vessels were the main route of tumor tissue transport to the blood. Generally, clinical application of exosome technology could lead to numerous new discoveries and procedures.

### Application Prospects of Exosomes in the Field of Cancer Chemoresistance

Exosomes have broad application potential in chemoresistance, including as biomarkers in liquid biopsy to monitor the development of chemoresistance, in the study of chemoresistance mechanisms, and in the development of new strategies for reversing chemoresistance.

Exosomes can be used as biomarkers in liquid biopsies to monitor whether cancer is resistant to chemotherapy. Traditional tissue biopsy is invasive and painful for patients, requiring repeated sampling, and hindering dynamic monitoring. The rapid development of liquid biopsy technology has paved the way for the continuous and accurate monitoring of cancer development. Because exosomes carry proteins, RNAs, and DNAs of the parent cells, they have become the “third carriage” in the field of liquid biopsy after ctDNA and CTC. Compared with ctDNA and CTC, exosomes have the advantages of comprising richer sample forms, providing more comprehensive information, harboring more stable contents, and having enormous potential and clinical application prospects. Nucleic acids and proteins in exosomes mediate the development of chemoresistance in cancer cells, and their differential expression are useful as a guide for cancer chemosensitivity, allowing clinicians to formulate a reasonable chemotherapy plan.

By exploring the mechanism of chemoresistance, new strategies for the reversal of cancer drug resistance can be developed. Exosomes, together with their nucleic acids and proteins, were previously found to mediate intercellular signal transduction and activate intracellular signaling pathways through fusion or interaction with target cells, including cancer cells. Following this approach, researchers can “take the essence and get rid of the dregs.” Liu T. et al. (2019) found that the expression of miR-128-3p was significantly lower in oxaliplatin CRC cells than in parent cells. *In vivo* and *in vitro* experiments have shown that miR-128-3p reduced chemoresistance of CRC to oxaliplatin. Subsequently, co-culture of drug-resistant CRC cells with exosomes successfully transfected with miR-128-3p increased the cells’ sensitivity to oxaliplatin (Liu T. et al., 2019).

In recent years, exosomes have opened up a new frontier for nano-delivery vehicles and also provided a new strategy for reversing chemoresistance due to their unique advantages (Tian et al., 2014; Logozzi et al., 2021). Compared to synthetic nanoparticles, exosomes have many advantages: (1) exosomes can overcome biological barriers due to their small size, and have high biocompatibility and cellular uptake ability due to the membrane protein (Mulcahy et al., 2014); (2) due to proteins and lipids, exosomes as a vectorized signaling device that appears more efficient than a soluble agonist (Huber et al., 2005); (3) exosomes also have high stability, tissue specific targeting, and drug delivery through exosomes can avoid phagocytosis or degradation of macrophages (Sun et al., 2010; Haney et al., 2015). Although exosomes have these advantages, they still face many challenges as nano-delivery vehicles. At the beginning of the research, the efficiency of the simple incubation method was too low and very limited in the type of cargo to be loaded. Various improvement strategies such as electroporation, transfection, and sonication have been developed for loading therapeutic cargoes into exosomes. Tian et al. (2014) showed that doxorubicin is loaded onto iRGD peptide functional exosomes by electroporation, and the drug loading efficiency is as high as 20-fold. Due to the presence of iRGD peptide, exosomes show high tumor targeting ability and anti-tumor efficacy in *in vitro* and *in vivo* experiments (Tian et al., 2014). Another major obstacle for the clinical translation of exosomes is their low yields. In most experimental studies, exosomes are obtained through cell culture. However, the obtained exosomes are difficult to meet the clinical needs. To overcome this limitation, some researchers have found that food contains a lot of exosomes (Del et al., 2021). Compared to cell culture, milk-derived exosomes showed a 1000-fold higher yield (Betker et al., 2019; Munir et al., 2020). There are also researchers who fused exosomes and liposomes to design hybrid therapeutic nanovesicles. Cheng et al. combined the characteristics of exosomes and thermosensitive liposomes, and demonstrated the potential of hybrid nanovesicles that block CD47 immune checkpoints in drug delivery in cancer treatment (Cheng et al., 2021). Therefore, although exosomes as nano-delivery vehicles have a bright future, it is still in its infancy, and further research is needed to achieve clinical application.

Compared with nucleic acids and proteins, exosomal lipids have not been extensively studied, even though they play an important role in tumorigenesis and development. Record et al. (2014) found that exosomes rich in prostaglandin participated in cancer immune escape and promoted tumor growth. After prostaglandin is taken up by cells, it promotes phospholipid metabolism and changes the fatty acid oxidation state of target cells, thereby promoting cancer cell migration (Record et al., 2014). Aung et al. (2011) found that lipids in exosomes induced rituximab resistance in B-cell lymphoma. Skotland et al. (2017a) demonstrated that lipids in exosomes could be used as biomarkers for the diagnosis of prostate cancer. Therefore, besides their use as biomarkers, exosome lipids have a strong potential as mediators of chemoresistance. More and detailed work are warranted for studying the role of exosomes in regulating chemoresistance via lipids.

## Conclusion

Chemoresistance is one of the main obstacles to successful cancer treatment. In recent years, exosomes have attracted attention for their role in cancer occurrence and development. They act by transmitting chemoresistance phenotypes and reducing intracellular drug concentration. The differential expression of nucleic acids and proteins in exosomes reflects the physiological and pathological conditions of cells. By the same token, exosomes could also be used to reverse chemoresistance and function as biomarkers to monitor drug resistance in cancer cells. However, our understanding of the role of exosomes in cancer chemoresistance remains limited. Furthermore, the study of exosomes faces huge challenges. As such, the successful transition of exosome research into clinical practice requires the continuous efforts of medical researchers and clinical staff.

## Author Contributions

JL, YL, and TF conceived the manuscript. JL wrote the manuscript and prepared the figures. NG, ZG, WL, BP, XD, YL, and TF revised the manuscript. All the authors read and approved the final manuscript.

## Conflict of Interest

The authors declare that the research was conducted in the absence of any commercial or financial relationships that could be construed as a potential conflict of interest.

## Publisher’s Note

All claims expressed in this article are solely those of the authors and do not necessarily represent those of their affiliated organizations, or those of the publisher, the editors and the reviewers. Any product that may be evaluated in this article, or claim that may be made by its manufacturer, is not guaranteed or endorsed by the publisher.
